# Identification and Biochemical Properties of Two New Acetylcholinesterases in the Pond Wolf Spider (*Pardosa pseudoannulata*)

**DOI:** 10.1371/journal.pone.0158011

**Published:** 2016-06-23

**Authors:** Xiangkun Meng, Chunrui Li, Chunli Xiu, Jianhua Zhang, Jingjing Li, Lixin Huang, Yixi Zhang, Zewen Liu

**Affiliations:** Key Laboratory of Integrated Management of Crop Diseases and Pests (Ministry of Education), College of Plant Protection, Nanjing Agricultural University, Weigang 1, Nanjing, 210095, China; Weizmann Institute of Science, ISRAEL

## Abstract

Acetylcholinesterase (AChE), an important neurotransmitter hydrolase in both invertebrates and vertebrates, is targeted by organophosphorus and carbamate insecticides. In this study, two new AChEs were identified in the pond wolf spider *Pardosa pseudoannulata*, an important predatory natural enemy of several insect pests. In total, four AChEs were found in *P*. *pseudoannulata* (including two AChEs previously identified in our laboratory). The new putative AChEs PpAChE3 and PpAChE4 contain most of the common features of the AChE family, including cysteine residues, choline binding sites, the conserved sequence ‘FGESAG’ and conserved aromatic residues but with a catalytic triad of ‘SDH’ rather than ‘SEH’. Recombinant enzymes expressed in Sf9 cells showed significant differences in biochemical properties compared to other AChEs, such as the optimal pH, substrate specificity, and catalytic efficiency. Among three test substrates, PpAChE1, PpAChE3 and PpAChE4 showed the highest catalytic efficiency (*V*_max_/*K*_M_) for ATC (acetylthiocholine iodide), with PpAChE3 exhibiting a clear preference for ATC based on the *V*_max_^ATC^/*V*_max_^BTC^ ratio. In addition, the four PpAChEs were more sensitive to the AChE-specific inhibitor BW284C51, which acts against ATC hydrolysis, than to the BChE-specific inhibitor ISO-OMPA, which acts against BTC hydrolysis, with at least a 8.5-fold difference in *IC*_50_ values for each PpAChE. PpAChE3, PpAChE4, and PpAChE1 were more sensitive than PpAChE2 to the tested Carb insecticides, and PpAChE3 was more sensitive than the other three AChEs to the tested OP insecticides. Based on all the results, two new functional AChEs were identified from *P*. *pseudoannulata*. The differences in AChE sequence between this spider and insects enrich our knowledge of invertebrate AChE diversity, and our findings will be helpful for understanding the selectivity of insecticides between insects and natural enemy spiders.

## Introduction

Acetylcholinesterase (AChE, EC 3.1.1.7) is an important neurotransmitter hydrolase in invertebrate and vertebrate nervous systems. AChE hydrolyses the neurotransmitter acetylcholine (ACh) and terminates nerve impulses at cholinergic synapses. Studies on AChE involvement in human diseases can lead to treatment approaches for some human disorders, such as Alzheimer's disease [1, 2]. In agricultural pest management, many insecticides, including organophosphorus (OP) and carbamate (Carb) insecticides, are designed to target AChEs. AChEs are encoded by *ace* genes in both invertebrates and vertebrates; however, the number of *ace* genes varies among species. Insects have been reported to possess one or two *ace* genes [3]. Only one *ace* gene is present in the genome of the spider mite *Tetranychus urticae* [4]. Nematodes carry four *ace* genes [5]. In contrast, more than four *ace* genes may exist in spiders because transcriptome annotation has identified multiple putative AChE unigenes in the *Pardosa pseudoannulata* transcriptome [6] and *Stegodyphus mimosarum* genome (GenBank Accession numbers: AZAQ00000000).

The pond wolf spider *P*. *pseudoannulata* is an important natural enemy of several insect pests and is widespread in the agricultural ecosystem of Asia [7]. Although OP and Carb insecticides show high toxicity toward insect pests, such as rice planthoppers, these compounds are relatively safe for predatory *P*. *pseudoannulata* [8, 9]. Target differences between insect pests and *P*. *pseudoannulata* partly contribute to insecticide selectivity. In our previous study, two AChEs (PpAChE1 and PpAChE2) were cloned from *P*. *pseudoannulata*, and these enzymes showed significantly different sensitivities to OP and Carb insecticides *in vivo* and *in vitro* [9, 10]. AChEs have been well studied in insects to date. AChE1 was determined to be the main catalytic enzyme in most insects, with most resistance mutations appearing in the *ace1* gene [3, 11–14]. In comparison, AChEs have rarely been studied in natural enemy spiders. Pharmacological studies of insecticide targets of insect pests and their natural enemies are essential for understanding insecticide selectivity and for the rational use of insecticides.

Here, we describe the gene cloning, bioinformatic analysis, Sf9 cell functional expression, and recombinant enzyme biochemical properties of two new AChE-encoding genes from *P*. *pseudoannulata*. In total, four AChEs were found in *P*. *pseudoannulata* (including two AChEs previously identified in our laboratory). These results will provide important information regarding the diversity and evolution of the spider AChE system and the selectivity mechanisms of insecticide targeting of AChEs between insects and natural enemy spiders, as well as guidance for integrated pest management.

## Materials and Methods

### Spiders, chemicals and Sf9 cell lines

*P*. *pseudoannulata* spiders were collected from paddy fields of Nanjing (Jiangsu, China, longitude/latitude: 118°35′/32°04′) in August 2014 and stored in liquid nitrogen before use. We confirmed that the location was not privately owned or protected in any way and that the species collection did not involve endangered or protected species.

The insecticide diazoxon (CAS 962-58-3, 99.0%) was purchased from J&K Scientific Ltd (Beijing, China). Paraoxon (CAS 311-45-5), carbaryl (CAS 63-25-2), fenobucarb (CAS 3766-81-2), eserine (CAS. 57-64-7), BW284C51 (CAS 402-40-4), ISO-OMPA (CAS 513-00-8), acetylthiocholine iodide (ATC, CAS 1866-15-5), butyrylthiocholine iodide (BTC, CAS 1866-16-6) and propionylthiocholine iodide (PTC, CAS 1866-73-5) were purchased from Sigma (St. Louis, MO, USA). Sf9 cell lines were purchased from Invitrogen (Carlsbad, CA, USA).

### *P*. *pseudoannulata* RNA extraction and *ace* gene cloning

Total RNA was extracted from a single female spider using the Trizol reagent (Invitrogen, Carlsbad, CA, USA). Rapid amplification of cDNA ends (RACE) was performed with 5' and 3' full RACE Core Set (TaKaRa, Dalian, China) according to the manufacturer’s instructions. Two putative *ace* genes in the *P*. *pseudoannulata* transcriptome were selected and cloned using RACE technology with an individual specific primer (S1 Table).

### Homology analysis of two new putative AChEs

To assess homology, two complete sequences were blast searched using the NCBI online services at http://www.ncbi.nlm.nih.gov/BLAST. Protein alignments were generated using Vector NTI 11.5 and GeneDoc 2.7 software. Phylogenetic relationships among AChEs were examined using MEGA 5.05 software. A phylogenetic tree was generated employing the neighbour-joining method, and the branch strength of the tree was evaluated via bootstrapping with 1000 iterations.

### Expression of two new putative AChEs in Sf9 cells

Sf9 cells were used to express two new putative AChEs, as well as two AChEs previously identified in our laboratory, and an enhanced green fluorescent protein (eGFP, GenBank accession number: AAK15492) using Bac-to-Bac systems. The complete coding regions of the five genes were subcloned into the pFastBac-HTa vector (Invitrogen, Carlsbad, CA, USA) at multiple cloning BamH I and Hind III sites using the ClonExpress II One Step Cloning kit (Vazyme, Nanjing, China) and individual specific primers (S2 Table) according to the manufacturer instructions; the results were verified by nucleotide sequencing. Sf9 cell culture, infection, and expression were performed as previously described [9]. Briefly, recombinant Bacmid DNAs were transfected into Sf9 cells to produce recombinant baculovirus, which was used for protein expression. The baculovirus culture supernatants were collected as the crude enzymes, and the culture supernatants of Sf9 cells not expressing AChEs were used as the negative control.

### Assay of enzyme biochemical properties

Enzyme activities were detected in a 96-well microplate with 100 μL of substrate at different concentrations, 50 μL of enzyme and 100 μL of dithiobis-(2-nitrobenzoic acid) (DTNB, 75 μM). The reactions were monitored for 20 min in 30 s intervals at 405 nm using a Molecular Devices Thermomax Kinetic Microplate Reader. At least three replications were used to calculate the mean level of enzyme activity. To determine the optimal pH condition of each enzyme, the pH of mixed-reaction solutions was adjusted using NaOH or HCl. Under the optimal pH, the Michaelis-Menten constant value (*K*_M_) and maximal reaction velocity (*V*_max_) at different substrate concentrations were evaluated for 2 min via nonlinear regression of enzyme activity measurements. For PpAChE1 and PpAChE2, the ATC, BTC and PTC substrate concentrations ranged from 20 μM to 2000 μM; for PpAChE3 and PpAChE4, the ATC concentrations ranged from 5 μM to 500 μM and the BTC and PTC concentrations ranged from 20 μM to 2000 μM. The Michaelis constant (*K*_M_) and maximum velocity (*V*_max_) for each substrate were determined by fitting the velocity (ν) and substrate concentration ([*S*]) data to *ν* = *V*_max_ [*S*]/ (*K*_M_ +[*S*]) using Prism 5.0 (GraphPad Software, La Jolla, CA, USA). Protein concentrations were measured by the Protein-dye Binding Method using bovine serum albumin as a standard.

The methods applied for inhibitors were performed as described previously [9,15]. To determine *IC*_50_ values, the enzymes were first incubated for 10 min with an inhibitor at various concentrations. The residual enzyme activity (in reference to a mixture of the enzyme and buffer) was determined as above and plotted against -log_10_[I]. The *IC*_50_ value for each inhibitor was determined from nonlinear regression analysis of activity and -log_10_[I] data using Prism 5.0 (GraphPad Software, USA). The bimolecular rate constant (*k*_i_) was determined by enzyme reaction premixed with various concentrations of an inhibitor and was fitted using Prism 3.0 (GraphPad Software, La Jolla, CA, USA).

### Data analysis

Pooled data are presented as the means ± SEM of at least five independent experiments. Statistical significance was determined by one-way ANOVA, together with Fisher’s least significant difference post-hoc test for pairwise comparisons. Data were considered to be significant at *p* < 0.05.

## Results

### Cloning and sequence analysis of two putative acetylcholinesterase genes from *P*. *pseudoannulata*

**Fig 1:**
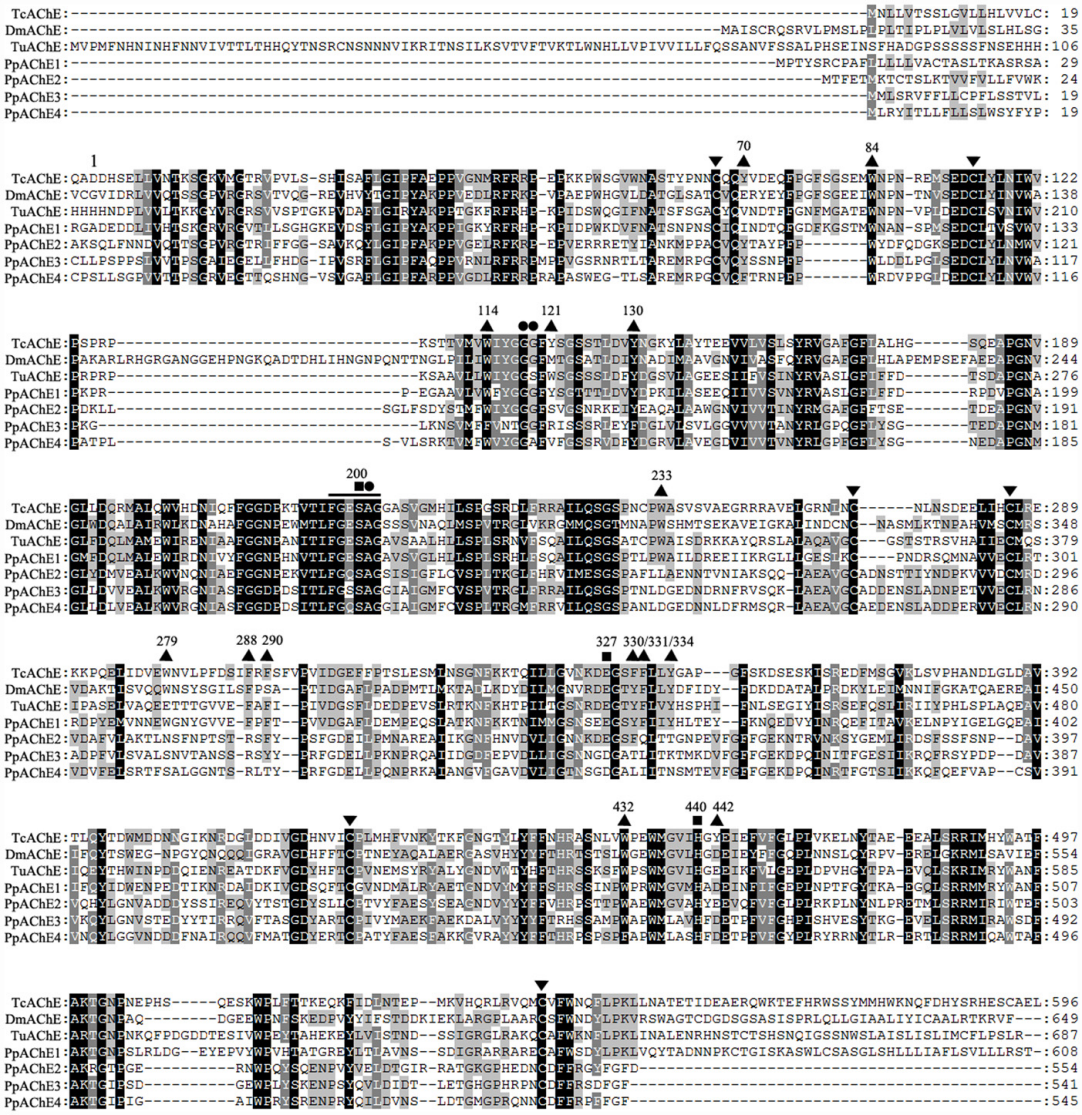
Amino acid sequence alignment of acetylcholinesterases from different species. Identical amino acids are shaded in grey for 80% similarity and black for 100% similarity. The ‘▲’ indicates the 14 aromatic residues, ‘▼’ shows the six cysteine residues, ‘■’ indicates the catalytic triads, and ‘●’ represents the oxyanion hole. The conserved sequence ‘FGESAG’ is underlined. The numbering on the amino acid sequences indicates the number for *T*. *californica* AChE amino acids, which starts at the N-terminus of the mature protein. Tc: *Torpedo californica* (CAA27169); Dm: *Drosophila melanogaster* (P07140); Tu: *Tetranychus urticae* (AAO73450); Pp: *Pardosa pseudoannulata* (KF543247, KU501286, KU501287, KU501288).

**Fig 2:**
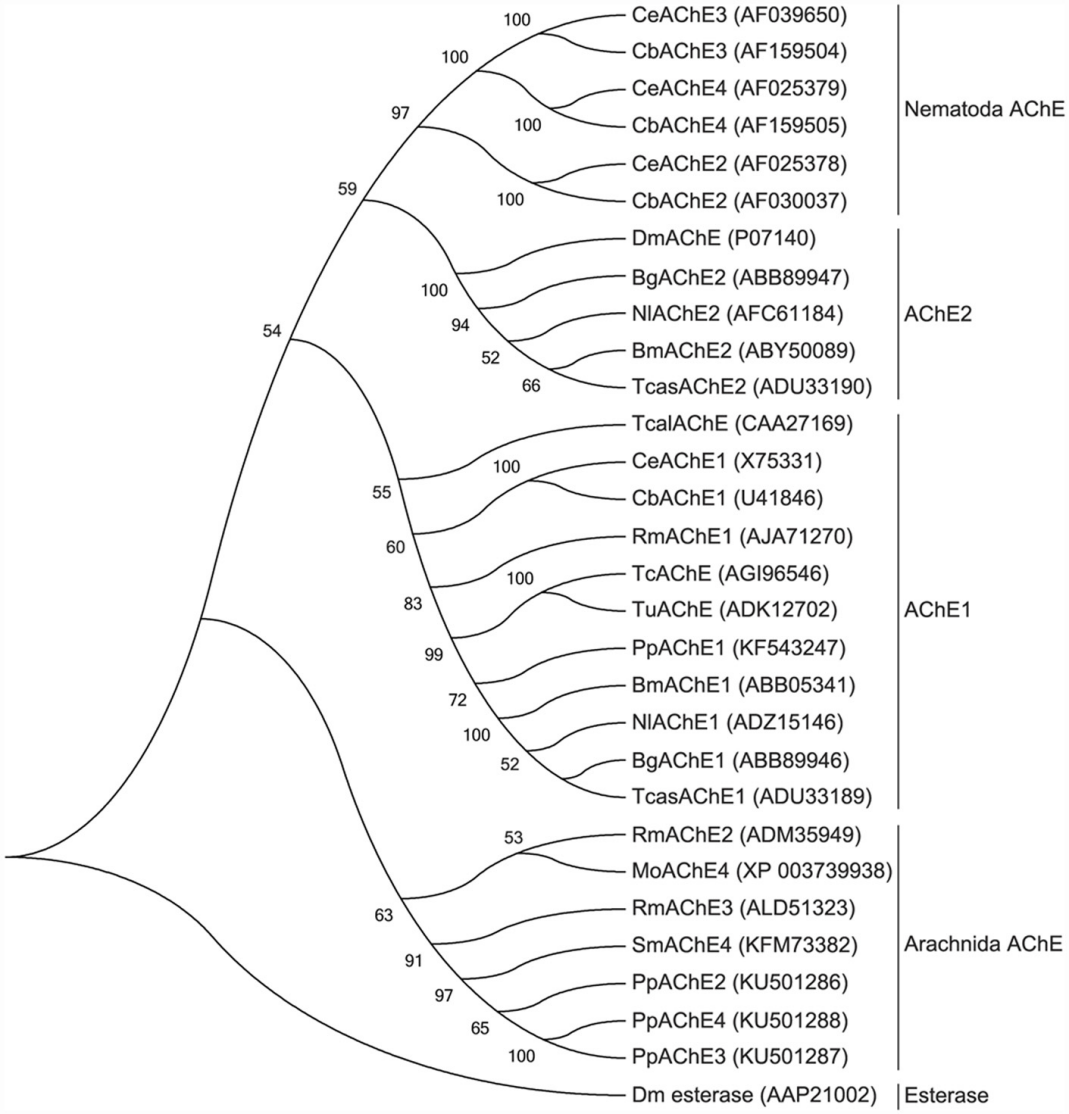
Phylogenetic analysis of two putative AChEs from *P*. *pseudoannulata* compared with AChEs from other species. Numbers above the branches indicate phylogenies based on amino acid sequences; only values above 50% are shown. Tcal: *Torpedo californica*; Dm: *Drosophila melanogaster*; Bg: *Blattella germanica*; Bm: *Bombyx mori*; Nl: *Nilaparvata lugens*; Tcas: *Tribolium castaneum*; Tc: *Tetranychus cinnabarinus*; Tu: *Tetranychus urticae*; Rm: *Rhipicephalus microplus*; Ce: *Caenorhabditis elegans*; Cb: *Caenorhabditis briggsae*; Mo: *Metaseiulus occidentalis*; Sm: *Stegodyphus mimosarum*; Pp: *Pardosa pseudoannulata*.

Several putative *ace* unigenes were identified in the *P*. *pseudoannulata* transcriptome [6], and two new full-length putative *ace* genes were obtained by RACE. Two of the four putative AChEs identified in *P*. *pseudoannulata*, PpAChE1 and PpAChE2 (GenBank Accession numbers: KF543247, KF543248), were previously cloned in our laboratory and have minor amino acid changes, such as in the catalytic triad in PpAChE2 (GenBank Accession number: KU501286) [9]. BLAST of the deduced amino acids shows 29.8%-40.5% identity with *Torpedo californica* AChE and 26.6%-32.8% identity with *Drosophila melanogaster* AChE (Fig 1). Based on these identities, the two putative AChEs were named PpAChE3 and PpAChE4 (GenBank Accession numbers: KU501287, KU501288). PpAChE3 and PpAChE4 possess the most common features of the AChE family, such as conserved cysteine residues, choline binding sites, and the conserved sequence ‘FGESAG’ but with an ‘SDH’ catalytic triad instead of ‘SEH’ (Fig 1). Some important amino acids, including aromatic residues, oxyanion holes, and catalytic triads, were compared among AChEs from *Torpedo*, insect, spider mite and *P*. *pseudoannulata* (Table 1). It was found that some, but not all, aromatic residues having important roles in AChE function are conserved in the putative AChEs from *P*. *pseudoannulata*. Phylogenetic analysis of the two new putative AChEs, as well as PpAChE1 and PpAChE2, with AChEs from other species was performed and clearly demonstrated that PpAChE1 is well classified with invertebrate AChE1; the other three are classified with Arachnida AChEs (Fig 2).

**Table 1:** Key amino acid differences at functional sites among AChEs of *T*. *californica*, *D*. *melanogaster*, *T*. *urticae*, and *P*. *pseudoannulata*.

Subsite	*T*. *californica*	*D*. *melanogaster*	*T*. *urticae*	PpAChE1	PpAChE2	PpAChE3	PpAChE4
Catalytic triad	S200	S	S	S	S	S	S
	E327	E	E	E	E	D	D
	H440	H	H	H	H	H	H
Oxyanion hole	G118	G	G	G	G	G	G
	G119	G	S	G	G	G	A
	A201	A	A	A	A	A	A
Choline binding site	**W84**	**W**	**W**	**W**	**W**	**W**	**W**
	**Y130**	**Y**	**Y**	**Y**	**Y**	**F**	**Y**
	**F330**	**Y**	**Y**	**Y**	**F**	T	L
	**F331**	**F**	**F**	**F**	Q	L	I
Acyl pocket	**F288**	**F**	**F**	**F**	R	R	R
	**F290**	S	**F**	**F**	**F**	**Y**	T
	V400	**F**	**F**	**F**	L	R	R
Peripheral anionic site	**Y70**	E	V	I	**Y**	**Y**	**F**
	**Y121**	M	**W**	**Y**	S	R	V
	**W279**	**W**	E	**W**	N	S	S
Wall of the gorge	**W114**	**W**	**W**	**W**	**W**	**F**	**W**
	**W233**	**W**	**W**	**W**	L	D	D
	**Y334**	**Y**	**Y**	**Y**	T	K	N
	**W432**	**W**	**W**	**W**	**W**	**W**	**F**
	**Y442**	D	E	D	E	D	D

Residues in *T*. *californica* are used as reference values. The numbering indicates the number for *T*. *californica* AChE amino acids, which starts at the N-terminus of the mature protein. Conserved aromatic residues are shown in bold type.

### Recombinant expression of four AChEs

Four AChEs from *P*. *pseudoannulata* were expressed in Sf9 cells using a baculovirus-insect expression system with enhanced green fluorescent protein (eGFP) as a visual control. The recombinant proteins were successfully expressed based on detection of fluorescence and virus-infected cell form (S1 Fig). The expressed proteins were collected for the analyses of biochemical properties.

**Fig 3:**
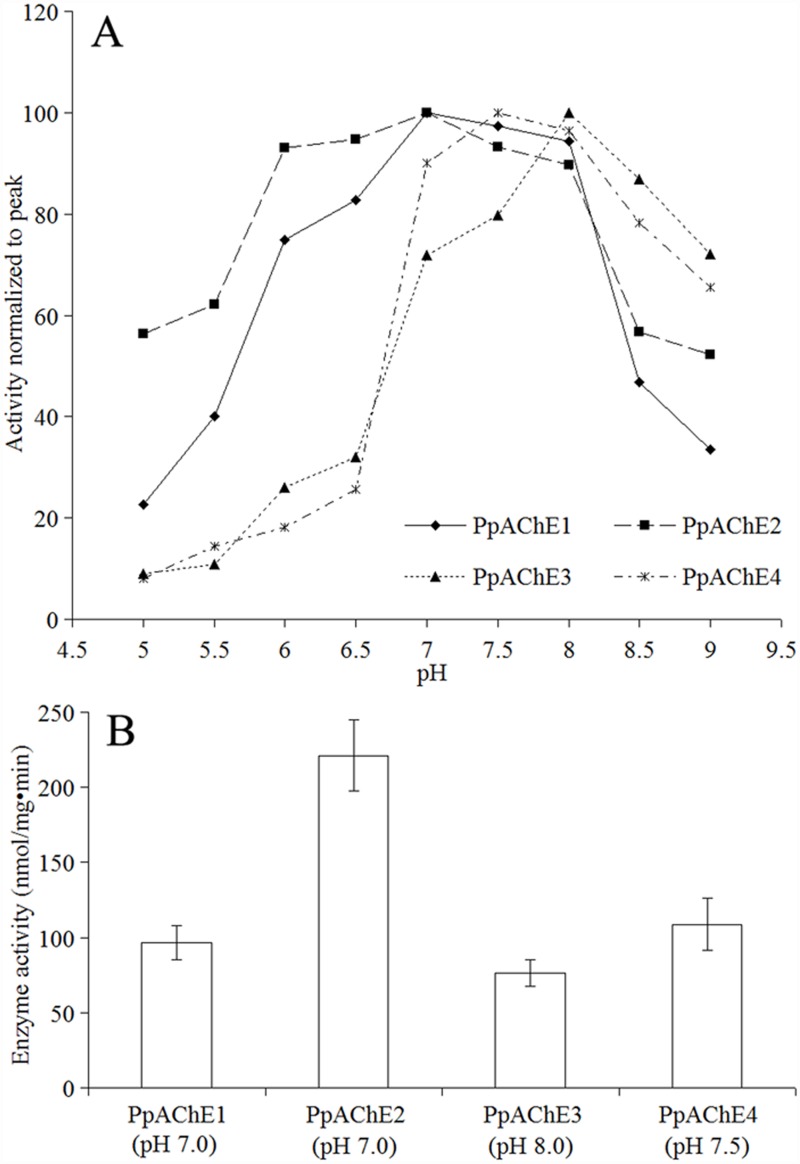
Optimal pH conditions for different recombinant enzymes. A: Enzyme activities under different pH conditions. B: Enzyme activities of four AChEs under their optimal pH conditions. Data are the mean±SEM of at least five independent experiments. For clarity, only one representative curve for each enzyme is shown.

The enzyme activities of the recombinant AChEs were measured at different times the after virus infection, and all enzymes were found to have maximum activity at 72 h after infection. Additionally, enzyme activity was tested at different pH values, and the results showed different optimal pH values for the recombinant AChEs. PpAChE1 and PpAChE2 exhibited maximum activities at pH 7.0, PpAChE3 at 8.0, and PpAChE4 at 7.5 (Fig 3A). Under the optimal pH condition for each AChE, PpAChE2 showed the highest activity, with a rate of more than 200 nmol/mg·min, than the other three AChEs (Fig 3B), which might be due to a higher level of expression in Sf9 cells.

### Substrate hydrolysis kinetics of recombinant AChEs

Four substrates were tested to identify the substrate preference of the recombinant AChEs. In the analysis of kinetic parameters, the Michaelis-Menten constant value (*K*_M_) and maximal reaction velocity (*V*_max_) of PpAChE1-4 were compared for ATC, butyrylthiocholine iodide (BTC) and propionylthiocholine iodide (PTC) (Table 2). PpAChE1, PpAChE3 and PpAChE4 had much lower *K*_M_ values for ATC than for the other two substrates, whereas PpAChE2 showed similar *K*_M_ values for all three substrates. In addition, PpAChE1, PpAChE3 and PpAChE4 showed the highest catalytic efficiency (*V*_max_/*K*_M_) for ATC, whereas PpAChE2 displayed similar catalytic efficiency for the three substrates. The *V*_max_ ratio was calculated between every pair of the substrates, and the high *V*_max_^ATC^/*V*_max_^BTC^ ratio of 3.1-fold for PpAChE3 indicated that PpAChE3 has a preference for ATC.

**Table 2:** Kinetics of substrate hydrolysis for four AChEs.

	*K*_M_ (μM)			*V*_max_ (nmol/mg•min)			*V*_max_/*K*_M_ (mL/mg•min)			*V*_max_ ratio		
	ATC	BTC	PTC	ATC	BTC	PTC	ATC	BTC	PTC	ATC *vs* BTC	ATC *vs* PTC	BTC *vs* PTC
PpAChE1	536.8±63.4c	858.8±103.2c	1148.6±133.5c	112.6±8.8b	95.4±12.8b	76.2±9.2c	209.8	111.1	66.3	1.18	1.48	1.25
PpAChE2	4413.5±533.7d	4542.2±326.0d	4496.3±318.8d	253.9±20.4a	233.9±16.5a	156.8±21.3a	57.5	51.5	34.9	1.09	1.62	1.49
PpAChE3	42.9±5.8a	198.6±22.3a	503.6±86.8b	124.7±11.5b	40.3±5.6c	106.2±15.1b	2906.8	202.9	210.9	3.09	1.17	0.38
PpAChE4	61.6±7.5b	269.4±35.5b	279.3±35.2a	86.6±9.7c	105.5±8.9b	49.7±8.4d	1405.8	391.6	177.9	0.82	1.74	2.12

Different lowercases in the same column indicate significant differences among the putative AChEs. The data are the mean±SEM of at least five independent experiments.

### Inhibition kinetics of recombinant AChEs

**Fig 4:**
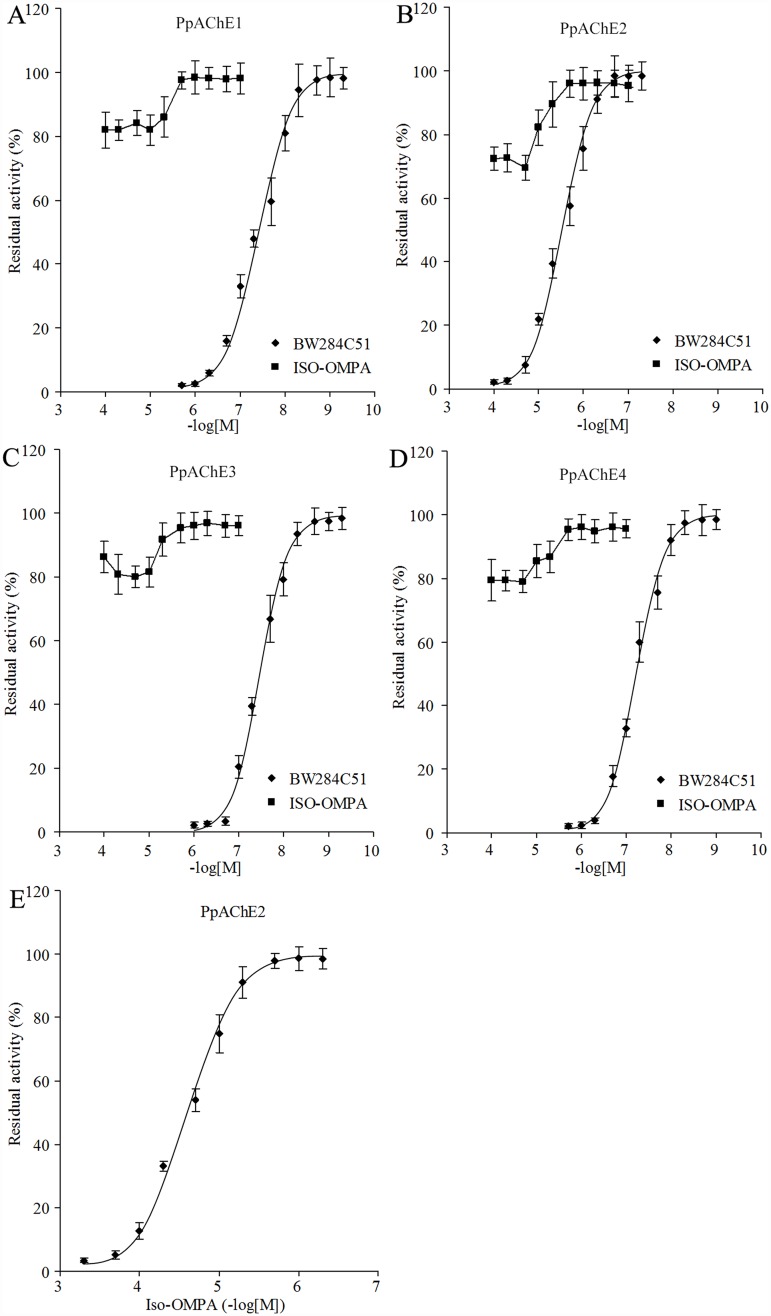
Inhibition curves of two inhibitors against four AChEs. A-D: Inhibition due to different doses of BW284C51 and ISO-OMPA against the ATC hydrolysis activity of four AChEs. E: Inhibition due to different doses of ISO-OMPA against the BTC hydrolysis activity of PpAChE2. The data are the mean±SEM of at least five independent experiments.

Inhibition of enzyme activity by three specific inhibitors was investigated for the four recombinant AChEs (Table 3). The cholinesterase-specific inhibitor eserine, acetylcholinesterase-specific inhibitor BW284C51, and butyrylcholinesterase-specific inhibitor ISO-OMPA were tested first. Both eserine and BW284C51 showed strong inhibition of the ATC hydrolysis activity of all recombinant AChEs, but the *IC*_50_ values for PpAChE2 were at least 7 times that of the other three PpAChEs (Table 3; Fig 4). In contrast to BW284C51, ISO-OMPA showed very weak inhibition against ATC hydrolysis by the four AChEs and could not completely inhibit ATC hydrolysis activity even at very high concentrations (Fig 4A–4D). ISO-OMPA showed slight inhibition of the BTC hydrolysis activity of the four AChEs, but much larger *IC*_50_ values were observed compared to the inhibition of ATC hydrolysis activity by eserine and BW284C51. ISO-OMPA showed an *IC*_50_ value less than 10^−4^ M only for PpAChE2 (Fig 4E), and the *IC*_50_ values for the other three AChEs were above 2×10^−4^ M. The exact values were not determined because of the high concentrations of ISO-OMPA used (Table 3). Analysis of the inhibition kinetics of the recombinant AChEs confirmed the differences in eserine sensitivities among the four PpAChEs, as PpAChE1, PpAChE3 and PpAChE4 showed much higher sensitivities to eserine than did PpAChE2 (Table 4).

**Table 3:** *IC*_50_ values for three inhibitors against enzyme activities of four AChEs (×10^−8^ M).

	On ATC hydrolysis		On BTC hydrolysis
	BW284C51	Eserine	ISO-OMPA
PpAChE1	5.12±0.76	11.67±2.06	>20000
PpAChE2	254.17±21.40	186.42±25.11	2165.39±325.67
PpAChE3	3.68±0.53	6.52±1.80	>20000
PpAChE4	7.36±1.05	24.50±3.94	>20000

**Table 4:** Inhibition kinetics (*k*_i_) of four AChEs (×10^−6^ M^-1^ min^-1^).

Compound	Eserine	Fenobucarb	Carbaryl	Paraoxon	Diazoxon
PpAChE1	9.52±0.63b	14.69±0.89a	1.13±0.05c	0.43±0.06c	0.13±0.05c
PpAChE2	0.83±0.10d	0.30±0.05d	0.12±0.03d	0.13±0.03d	0.08±0.03c
PpAChE3	13.04±1.07a	2.43±0.52c	3.62±0.44b	8.01±1.13a	3.55±0.32a
PpAChE4	6.33±0.48c	3.16±0.27b	5.88±0.92a	0.95±0.14b	1.13±0.18b

Different lowercases in the same column indicate significant differences among putative AChEs. The data are the mean±SEM of at least five independent experiments.

The ATC hydrolysis inhibitory activity of two OP and two Carb insecticides was also tested (Table 4). The four AChEs showed various sensitivities to the different insecticides. PpAChE2 was relatively resistant to all of the tested insecticides, though PpAChE1 and PpAChE4 were more sensitive to the two Carb insecticides (fenobucarb and carbaryl) and PpAChE3 to the two OP insecticides (paraoxon and diazoxon).

## Discussion

The number of AChE-encoding genes varies among invertebrates and vertebrates. AChEs have been extensively studied in insects, with most species having two AChEs and a few only one AChE (e.g., *D*. *melanogaster* and *Musca domestica*) [3, 16, 17]. Multiple AChE-encoding genes have been found in nematodes: three in *Bursaphelenchus xylophilus* [18] and four each in *Caenorhabditis elegans* and *Caenorhabditis briggsae* [5, 19]. Furthermore, three secretory acetylcholinesterases have been reported in the nematode *Nippostrongylus brasiliensis* and two in the lungworm *Dictyocaulus viviparus*. These results indicate that nematodes might harbour more than four acetylcholinesterases in their genomes [20–22]. In Arachnida, only one *ace* gene has been found in the spider mite *T*. *urticae*, and three have been biochemically demonstrated in the cattle tick *Rhipicephalus microplus* [4, 23]. Several acetylcholinesterase proteins are annotated for the spider *S*. *mimosarum* genome in the NCBI database, and 17 AChE unigenes have been identified in the *P*. *pseudoannulata* transcriptome [6].

Although only one AChE protein is present in vertebrates, the sister cholinesterase butyrylcholinesterase (BChE) appears to complement the function of AChE in neuromuscular junctions. Both AChE and BChE contain the common sequence features of the cholinesterase (ChE) family, such as cysteine residues, choline binding sites and catalytic triads [24, 25]. AChE and BChE share 51–54% sequence identity in mammals but exhibit distinct substrate and inhibitor specificities [26]. Although the aromatic residues lining the catalytic gorges of ChEs often provide a clear indication for distinguishing between vertebrate AChE and BChE [27], enzyme biochemical properties, such as substrate specificity and sensitivity to specific inhibitors, are essential for identification. AChE prefers the substrate ATC and is specifically inhibited by BW284C51. Conversely, BChE prefers the substrate BTC and is specifically inhibited by ISO-OMPA [28–31]. Because of the many AChEs in invertebrates, sequence analysis alone may be insufficient to reveal whether a ChE is an AChE or a BChE. Sequence analysis of two new putative AChEs from *P*. *pseudoannulata*, as well as two previously identified AChEs from this species, showed that all identified AChEs contain many crucial motifs of the AChE family, including six cysteine residues forming three intramolecular disulphide bridges, choline binding sites, catalytic triads functioning as a charge-relay system, and a number of aromatic residues lining the catalytic gorge [32, 33]. However, some key differences were found among the *P*. *pseudoannulata* PpAChEs. The catalytic triads (S200, E327 and H440 in *T*. *californica*) are essential for substrate hydrolysis. However, a D327 motif, instead of E327, is present in PpAChE3 and PpAChE4. Although this substitution has not been previously reported for AChEs, it has been reported for some esterases, such as cholesterol esterase, lipase, cocaine esterase, arylacetamide deacetylase and carboxylesterase, among which catalytic triads are also conserved [34, 35]. Of the four putative *P*. *pseudoannulata* AChEs, only PpAChE1 has the F288 residue; PpAChE4 does not have an aromatic residue at the 290 position, which is thought to be typical of many invertebrate AChEs and crucial for ATC hydrolysis [36, 37]. The ‘FGESAG’ sequence structure around the active serine residue has been reported to be slightly altered in different species. A modified ‘VGESAG’ is found in *C*. *elegans* and *C*. *briggsae* ACE-3 [5]. ‘FGQSAG’ is detected in *C*. *elegans* and *C*. *briggsae* ACE-4 as well as in PpAChE2 and PpAChE4 in this study [5]. The ‘FGESSG’ motif is present in *Plutella xylostella* AChE1 and PpAChE3 [38] and ‘FGWSAG’ and ‘VGQSAG’ in *R*. *microplus* AChE2 and AChE3, respectively [39]. Among the five conserved aromatic residues lining the catalytic gorge, four are present in PpAChE1, but only two are present in the other three PpAChEs. The impact of these differences in important amino acid residues on the activity and biochemical properties of the enzyme requires further study.

Sequence analysis might not be sufficient for a conclusion, and additional evidence such as substrate specificity and sensitivity to specific inhibitors is needed to confirm whether the enzymes are in fact AChEs. According to kinetics analysis, PpAChE3 and PpAChE4 exhibit higher efficiency (based on *V*_max_/*K*_M_ values) for ATC hydrolysis than that for hydrolysis of BTC or PTC. Furthermore, the ratio of *V*_max_^ATC^/*V*_max_^BTC^ for PpAChE3 showed a clear ATC substrate preference. All four AChEs were more sensitive to the AChE-specific inhibitor BW284C51, which acts against ATC hydrolysis, than to the BChE-specific inhibitor ISO-OMPA, which acts against BTC hydrolysis, with at least a 8.5-fold difference in *IC*_50_ value between BW284C51 and ISO-OMPA for a given PpAChE. Taken together, these data support the conclusion that PpAChE3 and PpAChE4 are functional acetylcholinesterases in *P*. *pseudoannulata*. To further characterize the biochemical properties of new acetylcholinesterases, purification and analyses of the purified enzymes are important and will performed in the near future.

One compelling question is why do spiders possess so many acetylcholinesterases. Multiple AChEs in a given species can show different biochemical properties, such as the nematodes *C*. *briggsae* and *B*. *xylophilus*, the tick *R*. *microplus*, and many insects [3, 5, 23]. The four AChEs from *P*. *pseudoannulata* share identities between 27.7% and 63.4%, and these sequence differences may contribute to different enzyme properties. Phylogenetic mapping of the four PpAChEs with other arthropod AChEs clearly showed that PpAChE2-4 are closely related to each other, indicating that they might have evolved from a common orthologous gene. The generation of distinct multiple AChE2 isoforms may occur via gene duplications and alternative splicing and be followed by divergent structural and functional evolution [3]. Phylogenetic analysis suggested that PpAChE2-4 may have developed from a single *ace2* locus and then evolved via gene duplication. Acetylcholinesterase is an ancient enzyme and is found in plants, bacteria as well as invertebrates and vertebrates [40]. In addition to the classical synaptic catalytic function, a variety of AChEs non-synaptic functions have been determined [41–44]. The four AChEs in *P*. *pseudoannulata* may have different functions, and the physiological function of each PpAChE remains to be elucidated.

The sensitivities of the four AChEs to different insecticides were significantly different according to *k*_i_ values. In our previous studies, the sensitivities of PpAChE1 and PpAChE2 to different insecticides were validated *in vivo* and *in vitro*, showing considerably different sensitivities to OP and Carb insecticides [9, 10]. In the present study, we found that PpAChE1, PpAChE3 and PpAChE4 were more sensitive to Carb insecticides (fenobucarb and carbaryl) than PpAChE2 and that PpAChE3 was more sensitive to OP insecticides (paraoxon and diazoxon) than the other three AChEs. The results suggested that OP and Carb insecticides may have different target AChEs in *P*. *pseudoannulata*, even though the targets of these two classes of insecticides are in fact AChEs.

In conclusion, we report the identification of two new functional AChEs in the spider *P*. *pseudoannulata*, findings that increase the number of known AChEs in this spider to four. The biochemical properties of the four PpAChEs indicate diverse enzyme activity and inhibitor sensitivity, including different sensitivities to insecticides. An understanding of the differences in AChEs as an insecticide target will require knowledge of the molecular basis of the kinetic and pharmacological properties of these enzymes.

## Supporting Information

S1 Fig Fluorescence of Sf9 cells expressing enhanced green fluorescent protein (eGFP) at different times after infection. (TIF)

S1 Table Specific primers used for gene amplification. (DOC)

S2 Table Specific primers used for gene expression. (DOC)
