# Supplementary figures and images for: Identification and Biochemical Properties of Two New Acetylcholinesterases in the Pond Wolf Spider (Pardosa pseudoannulata)
Source: PLoS One. 2016 Jun 23;11(6):e0158011. doi: 10.1371/journal.pone.0158011 (PMC4919072; doi:10.1371/journal.pone.0158011)

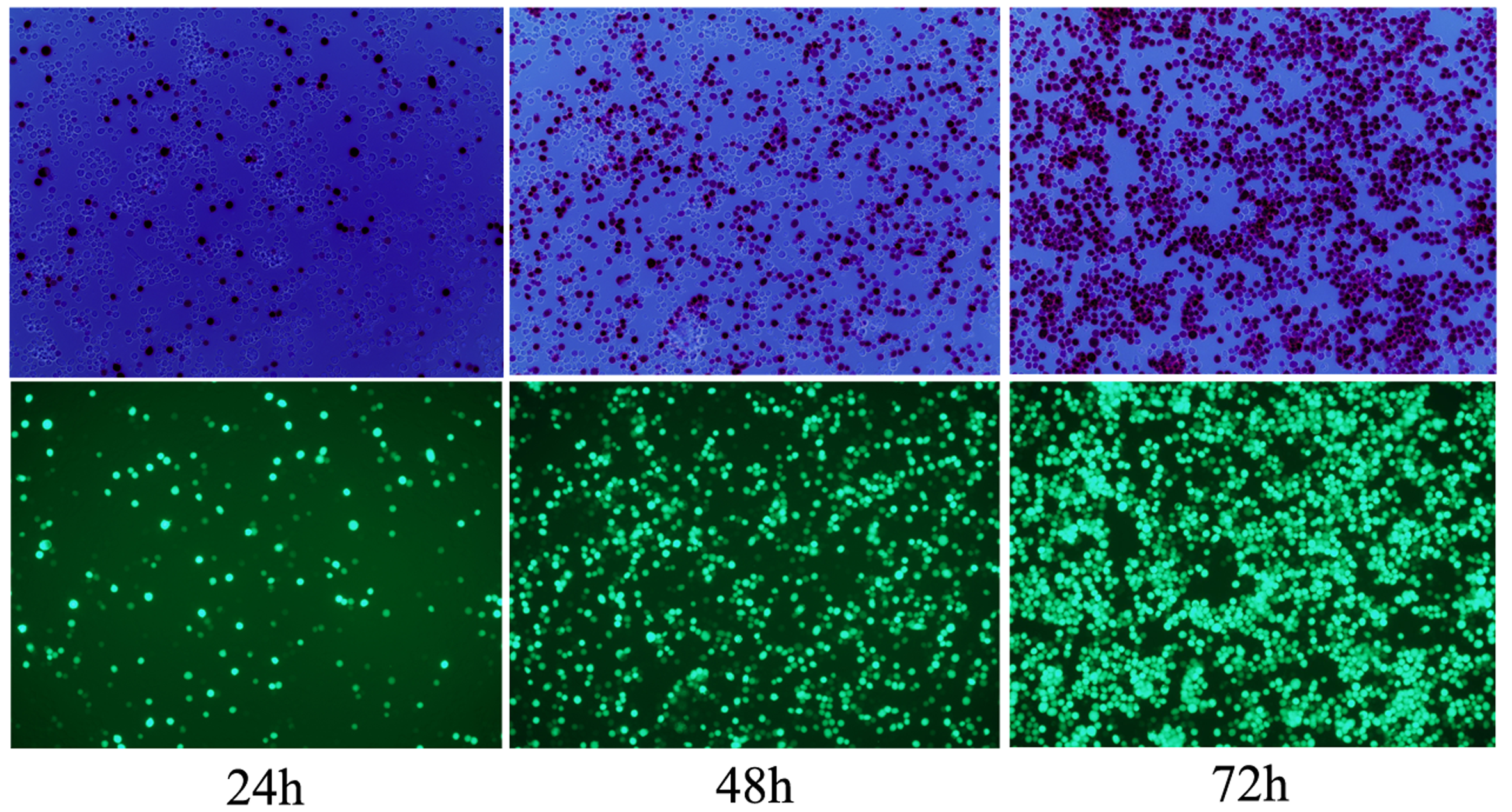

Supplement: S1 Fig — (TIF) [file pone.0158011.s001.tif]
