# Supplementary material for: Identification and Biochemical Properties of Two New Acetylcholinesterases in the Pond Wolf Spider (Pardosa pseudoannulata)
Source: PLoS One. 2016 Jun 23;11(6):e0158011. doi: 10.1371/journal.pone.0158011 (PMC4919072; doi:10.1371/journal.pone.0158011)
Supplement: S1 Table — (DOC) [file pone.0158011.s002.doc]

**S1 Table**. Specific primers used for gene amplification.

| Genes | 3` RACE primers | | 5` RACE primers |
| --- | --- | --- | --- |
| PpAChE3 | Outer primer: CACAGCCTCTGGAGATTATGCG | Outer primer: TACTGCTTTACGACGGCATCTGG | |
|  | Inner primer: TCGTGTTCGGACACCCCATCAG | Inner primer: CTGGTGACAGGCGATACGCAGAA | |
| PpAChE4 | Outer primer: GCGACTGGGGACTACGAAAGGA | Outer primer: TCAAGGGATAGCCGAACACGAA | |
|  | Inner primer: CCCTTCGTGTTCGGCTATCCCT | Inner primer: CACCAAAGTCGCCGTTCGCAAT | |

GenBank accession number: PpAChE3 (KU501287), PpAChE4 (KU501288).
