# Supplementary material for: Identification and Biochemical Properties of Two New Acetylcholinesterases in the Pond Wolf Spider (Pardosa pseudoannulata)
Source: PLoS One. 2016 Jun 23;11(6):e0158011. doi: 10.1371/journal.pone.0158011 (PMC4919072; doi:10.1371/journal.pone.0158011)
Supplement: S2 Table — (DOC) [file pone.0158011.s003.doc]

**S2 Table**. Specific primers used for gene expression.

| Genes | Expression primers |
| --- | --- |
| PpAChE1 | Sense primer: TAGTGCGGCCGCTTTCGAATATGCCTACGTACAGCCGATG |
|  | Anti-sense primer: CTCGAGACTGCAGGCTCTAGCTACGTAGACCTAAGAAGAA |
| PpAChE2 | Sense primer: TAGTGCGGCCGCTTTCGAATATGACTTTTGAAACGATGAA |
|  | Anti-sense primer: CTCGAGACTGCAGGCTCTAGCTAATCGAACCCAAAGTATC |
| PpAChE3 | Sense primer: TGTATTTTCAGGGCGCCATGATGATGCTTTCCAGAGTTTT |
|  | Anti-sense primer: CTCTAGTACTTCTCGACAATCATTAAAATCCAAAGTCGGAT |
| PpAChE4 | Sense primer: TAGTGCGGCCGCTTTCGAATATGCTGAGATATATTACTTT |
|  | Anti-sense primer: CTCGAGACTGCAGGCTCTAGTTAAAATCCGAAGAAGGGTC |
| eGFP | Sense primer: TAGTGCGGCCGCTTTCGAATATGGTGAGCAAGGGCGA |
|  | Anti-sense primer: CTCGAGACTGCAGGCTCTAGTTACTTGTACAGCTCGTC |
